# Understanding access to professional healthcare among asylum seekers facing gender-based violence: a qualitative study from a stakeholder perspective

**DOI:** 10.1186/s12914-020-00244-w

**Published:** 2020-09-21

**Authors:** Mirjam D. Rodella Sapia, Tenzin Wangmo, Stéphanie Dagron, Bernice S. Elger

**Affiliations:** 1 Institute for Biomedical Ethics, University of Basel, Basel, Switzerland; 2 University of Applied Sciences and Arts of Southern Switzerland, Manno, Switzerland; 3 Global Studies Institute / Institute of Global Health, University of Geneva, Geneva, Switzerland; 4 Center of Legal Medicine, University of Geneva, Geneva, Switzerland

**Keywords:** Gender-based violence, Women asylum seekers, Access to healthcare, Legal framework, women’s rights

## Abstract

**Background:**

When it comes to gender-based violence (GBV), migrant women and girls represent the most vulnerable group. GBV can happen at any stage of migrants’ flight and/or during the asylum process. It has severe consequences on their life and health. Victims therefore need timely access to healthcare. This study explores the context GBV victims face when they seek refuge in Switzerland.

**Methods:**

Qualitative methodology was used where we conducted five semi-structured focus groups and three interviews. A total of sixteen stakeholders participated in the study. They were either involved in the asylum process or provided healthcare to asylum seekers. We analyzed the data using framework analysis.

**Results:**

Study participants noted lack of confidence of the GBV victims in the legal and in the healthcare systems as major barriers to disclosure of GBV. Since only GBV exerted before fleeing the home country gives the right to asylum, they pointed out that victims do not disclose GBV that took place after they left their home country. Language was identified as a barrier to disclosure of GBV as well as to healthcare access. Continuity of care at the moment of transfer from federal to cantonal (i.e. state) accommodations is another issue that was deemed critical. Study participants felt that health professionals must be trained to identify GBV victims. The first-contact caregiver available to these victims was deemed as the most competent professional that could act as a “GBV coordinator”.

**Conclusion:**

In Switzerland, access to healthcare is guaranteed to all asylum seekers on a legal and structural level. Yet, health seeking by GBV survivors is hindered by factors such as lack of confidence in the legal system, trust in health providers, and continuity of care during the asylum process. Building trust in legal institutions, health structures, and professionals should be enhanced to facilitate disclosure and to strengthen resilience. This includes a healthcare system with competent professionals, support with language and cultural needs, as well as seamless continuity of care beyond cantonal borders.

## Background

The Convention on the Elimination of All Forms of Discrimination against Women (CEDAW) defines gender-based violence (GBV) as “violence that is directed against a woman because she is a woman or that affects women disproportionately. It includes acts that inflict physical, mental or sexual harm or suffering, threats of such acts, coercion and other deprivations of liberty” [3]. GBV is a human-rights violation shown to be associated with long-term health effects [6–9, 50]. It affects 35% of women worldwide [46]. The most reported is sexual assault, although GBV also includes physical, emotional and psychological violence, sex trafficking, forced prostitution, and harmful practices such as genital mutilation and forced marriage [41, 46]. Intimate-partner violence, as a form of GBV, is highest in the Eastern Mediterranean region (37.0%) and South-East Asia (37.7%). A recent study, collecting data from 22 countries of the Arab league, reveals prevalence ranging from 6 to 59% for physical abuse, from 3 to 40% for sexual abuse and from 5 to 91% for emotional/psychological intimate-partner violence [12]. Another recent publication highlights similar results in 27 sub-Saharan Africa countries [26]. In high income countries the rate of GBV is up to 23.2% [46]. In Switzerland, it is reported that one out of five women has experienced psychological or physical domestic violence at least once in her life [17].

GBV has severe consequences on the life and health of the survivors such as psychological suffering (e.g. anxiety, depression, alcohol misuse, smoking) and negative physical outcomes (e.g. injury, disease, shock, infection, or chronic diseases like high blood pressure or disabilities) [17, 31, 41]. GBV can lead to disease related mortality (e.g. injuries, AIDS, unsafe abortions) or even homicide and suicide. Early intervention can improve depression, anxiety, post-traumatic stress syndrome and functional impairment [37, 41, 46, 49].

During emergency situations, including fleeing one’s country, sexual violence is the most frequent form of GBV [42–44, 48], although other types of violence may be the reason for the flight or experienced on the migration route. It is estimated that more than 67% of migrant women and girls from Syria have been victims of at least one kind of GBV [11, 23, 39, 42, 43, 47, 48]. A few recent studies describe a prevalence of GBV on migration routes or in humanitarian settings [11, 23, 39, 42, 43]. Other studies report that GBV may also happen in asylum facilities [21, 27, 29]. Despite its occurrence, GBV often remains neglected and underreported due to fear of stigma, shame, and victimization [11, 40, 42, 43]. A report by the World Health Organization (WHO) underlined the limited and uneven coverage of services needed by survivors and limited availability of trained health workers [47, 48]. Considering these facts, GBV needs to be addressed in the country of origin, on the migration route as well as in the host country.

Switzerland is among the many host countries that receive asylum seekers, but only one report exists that discusses access to healthcare in this context [32]. This report underlines the need to address asylum seekers’ psychological distress once they have been transferred from a federal asylum center (first accommodation center) to the final destination. There is no specific mention of healthcare services for GBV victims [32]. Yet, early intervention has been proven to be effective in reducing psychological and physical consequences in the short and long term [37, 49]. There are several reasons why the care needs of this group often remain unnoticed and unaddressed. First of all, survivors of GBV are often unaware that services are available, or they do not seek these services fearing shame and blame [21, 23, 26, 29]. Furthermore, they lack the necessary language proficiency to access healthcare [1, 20]. Health professionals, for their part, may not always have the competencies to recognize the specific needs and may lack training and knowledge to identify vulnerable individuals or groups [23]. Barriers must therefore be minimized on both sides to ensure careful and correct access to needed care. To our knowledge, no research has been carried out that analyses access to professional healthcare among women asylum seekers who either are survivors of GBV or may be facing gender-based violence in host countries such as Switzerland and similar welfare states in Europe.

The 2030 Agenda for Sustainable Development, implemented in Switzerland [14, 15], sets out 17 Sustainable Development Goals with 169 targets. In particular, Goal 5 aims to achieve gender equality and empower women and girls, focusing on the elimination of all forms of discrimination and violence against them [45]. The CEDAW Committee in the periodic report of Switzerland highlighted the positive aspects in undertaking legislative reforms like the Federal Act on Measures against forced marriages in 2013, and the adoption of Article 124 of the Criminal Code, prohibiting female genital mutilation. Yet, the visibility and the implementation of the Convention across cantons (i.e. states) and communities has not been sufficiently addressed [4, 5]. Furthermore, it is “still concerned about underreporting of GBV in Switzerland, low prosecution and conviction rates, resulting in impunity for the perpetrators, the lack of a national action plan and the insufficient number of shelters” [4, 5]. One major issue in Switzerland is the political framework associated with 26 different cantons. Cantonal differences cause disparities in funding for shelters and a lack of support for NGOs working with the victims. The CEDAW Committee therefore recommended that Switzerland enhance its efforts in combating GBV by increasing its reporting, adopting a national action plan to strengthen services, and ensuring that medical professionals become more culturally sensitive and address linguistic barriers [4, 5].

In 2002, the Swiss Federal Office of Public Health (FOPH) launched the National Program on Migration and Health (2002–2007, 2008–2013, 2014–2017). The report underlines that causes of health inequality are migration-related factors such as traumatic experiences, exposure to violence, poor healthcare in the country of origin, discrimination, uncertain residence status, and poor knowledge of the national languages [18]. Furthermore, the report identifies the socio-economic situation, the lack of knowledge and poor health literacy as healthcare access barriers. The main objectives of the program are to lower disparities in healthcare access, to enhance migrant-oriented health promotion and prevention, to educate healthcare staff, and to include intercultural interpreting as a resource [18]. The report about healthcare in federal asylum centers contains some guidelines about how to approach different health related problems, but none specifically targeted at victims of GBV [32].

### Swiss context

Switzerland is a country with 8.5 million people, 25.1% of which are deemed to be of migrant background [16]. This number includes asylum seekers as well. An asylum seeker is a person seeking safety from persecution or serious harm in the country other than his or her own country [25]. Asylum seekers are protected in a temporary manner in accordance with the 1951 United Nations Convention. In 2018, Switzerland received 15,255 new asylum applications (2017: 18,088; 2016: 27,207). Most asylum seekers stem from Eritrea (*n* = 2825), followed by Syria (*n* = 1393), Afghanistan (*n* = 1186), Turkey (*n* = 1005) and Georgia (*n* = 873) [35, 36]. In that same year Switzerland was processing totally 62,050 asylum applications, of which 39.4% were women [35, 36].

Healthcare professionals in Switzerland must therefore be aware of and address thoroughly the care needs of GBV victims in this population, as well as offer access to care for all types of health consequences. A few studies have been carried out in Switzerland exploring asylum seekers within the healthcare system [1, 20, 24]. However, none have discussed the experiences of female asylum seekers who have faced GBV.

### Study purpose

It is imperative that all involved stakeholders, including social workers and healthcare professionals, screen for GBV in a sensitive way with a focus on appropriate communication, and obtain reliable data while protecting the confidentiality of victims. The aim of this study is to analyze the *context* that asylum seekers, who are GBV victims, face when seeking professional help in Switzerland. On the one hand, the study focuses on the organizational framework and on the current strategies for handling healthcare support. On the other hand, it tries to identify access barriers and it maps needed support.

## Methods

Qualitative methodology has been chosen because of the explorative nature of the study. We employed semi-structured interview guides for our focus-groups (FG) and individual interviews (I). The study was conducted in a border canton of Switzerland, one of the main entry points for asylum seekers.

### Participant recruitment

The study participants included stakeholders who are providing different types of care and services for asylum seekers including GBV survivors. Since we wanted to analyze the *context*, we interviewed stakeholders who work with asylum seekers. The stakeholders included those who support asylum seekers from their arrival at the entry point to Switzerland to the first federal accommodation center, until the moment the asylum seeker has been transferred to a canton. We distinguished two levels of professionals among those who participated in the study: (1) **Institutional level**: Governmental offices, NGOs with governmental mandates and universities; (2) **healthcare level**: Physicians, nurses, first-contact caregiver and intercultural interpreters (see Table 1). The type of data collected (FG or I) was dependent on the professional setting of the interviewed person (team-work or individual).

**Table 1:** Study Participants

Government and Institutes		Number (characteristics) of participants
Focus Group 1	State Secretary of Migration	3 (non-HCP)
Focus Group 2	Federal Asylum Center	3 (2 HCP, 1 non-HCP)
Focus Group 3	NGO 1; NGO 2	2 (non-HCP)
Focus Group 4	University researcher	2 (non-HCP)
**Healthcare professionals**		
Focus Group 5	Physicians	3 (HCP)
Interview 1	Physician	1 (HCP)
Interview 2	Intercultural interpreter	1 (non-HCP)
Interview 3	First-contact caregiver	1 (non-HCP)
**Total**		**16**

*FG* Focus group; *I* Interview; *HCP* Healthcare professional; *non-HCP* non-Healthcare professional

Participants were selected by snowball sampling and were approached either by phone or by email. All contacted persons agreed to participate in the study. In total, 16 stakeholders participated in five focus groups and in three individual interviews. The age of the participants ranged from 32 to 65 years. Thirteen of them were women and three were men. The focus groups were homogeneous regarding the participants’ professional background except FG 2, which included a non-healthcare professional, a nurse, and a physician. In the result’s section, we use the distinction between healthcare professionals (HCP) and non-healthcare professionals (non-HCP) to guarantee anonymity of the participants. No further identifying information than that is given.

All data collection took place in the native language of the canton. Data collection stopped when it was clear to the interviewer that no new information was being gathered and that there were no other participants that could be contacted who fit the study purpose.

### Data collection and analysis

Data collection was carried out by the first author, a general physician and public health worker, who had experience with qualitative research. There was no professional involvement with the participants at the time of the study. The interviews took place at the workplace of the participants between October and November 2016. All the focus groups and interviews (except one interview) were audio taped and transcribed ad verbatim. Only one participant (HCP, I1) asked to switch off the recorder to feel more comfortable to report sensitive facts but allowed the interviewer to take notes. The interviews followed a semi-structured guide, which was created for the purpose of the project after studying preexisting guidelines and reports [23, 49]. The interview guide included questions within the framework of disclosure of GBV; identification and access to healthcare for GBV survivors; and prevention and strengthening GBV survivors’ resilience. More focused questions dealt with health problems (physical and mental), the communication of symptoms, and coping strategies. New inputs that emerged during an interview or focus group led to new themes to investigate. The interviewer therefore adapted the contents in the following focus group or interview considering these new aspects. The duration of the focus groups was between 40 and 60 min and the interviews lasted between 45 and 85 min. A total of 440 min of data were recorded.

Two coders carried out data analysis using Microsoft Word. A 5-step-framework analysis by Ritchie and Spencer (1994) was used: (1) familiarization with the data, (2) identification of thematic framework, (3) indexing or coding, (4) charting and mapping and (5) interpretation (as described in [2, 19]). After becoming familiarized with the interviews, we identified major themes at the level of individual and structural (legal and healthcare systems) aspects. Further, we indexed identified codes, charted them as keywords and mapped them within the four themes. The authors agreed on the categorization of the keywords into different themes and with the interpretation of the results presented in the text.

The cantonal ethics committee reviewed the study and deemed it exempt as it was outside of Switzerland’s Human Research Act. However, written informed consent was obtained from all participants after they were informed about the study orally and in writing.

## Results

### Confidence in the legal and healthcare systems

Confidence was noted as a strongly individual factor that needs to be addressed through targeted political and structural interventions. For the study participants, a GBV victim’s lack of confidence in the legal and in the healthcare systems represented a major barrier to disclosure and a hurdle to accessing healthcare facilities. The participants noted that the asylum seekers’ lack of confidence was due to their undecided political status in the host nation and to the uncertainties resulting from the migration process. Asylum seekers transit through countries where they live in illegality putting them at different risks (sexual assault, prostitution, exploitation, human trafficking, etc.) due to their vulnerable situation. In light of their past experiences in the home country and during flight, it is difficult for asylum seekers to believe that the host country will give protection and assure a legal status. They are thus suspicious of the legal system and its professionals. Underlining this point, a participant noted *“they come out of illegality and suddenly they should trust people and the state and believe us when we say that they live legally in our country*” (non-HCP, FG1). The same participants further said that “*they [the asylum seekers] often are unaware of their rights or are frightened of the [legal] system*”. Therefore, the ability to establish confidence between the host country and the asylum seekers represents a major issue.

Confidence in the healthcare system represented another important issue. Different cases had been reported where women asylum seekers, transiting through Egypt, had undergone a gynecological examination. Many of these young women did not know they received an intrauterine device (IUD). Those who knew were conscious about the risk of becoming victim of sexual violence and exploitation along their journey. A healthcare professional (FG5) observed that *“almost all young women who transited through Egypt have an IUD. I can recognize that it is not from here because the thread is different. They have all been to a clinic in Egypt. Some of them know, but many of them don’t know about the IUD”*. Such an encounter creates mistrust in the healthcare system as a medical intervention was carried out without the asylum seekers’ knowledge and consent. Host countries should therefore establish a safe environment that respects the patient’s right to self-determination. This way, victims of GBV can build confidence with the host country’s legal and healthcare systems. This could favor early disclosure and help seeking.

Disclosure of GBV is a very intimate decision and needs preparedness on the part of the victim. Several participants pointed out that to be able to work with GBV victims, they have to disclose sensitive details, or they have to be identified as victims. A barrier to disclosure was the fact that GBV experienced on the migration route does not affect the political status:

“GBV during migration route does not give the right to asylum. If they don’t want to stay here [the host nation they are either in or they are travelling through], they don't tell you about [this]. ( … ) Only GBV that has been reported in the country of origin and that is a reason for flight is important for asylum decisions” (non-HCP, FG1).

Thus, victims of GBV should know about their right to have access to specific healthcare independently of the influence on the asylum process. One participant underlining this point noted that *“often an unclear political status, [i.e. whether they will be recognized as asylum seekers or become a “non-entry decision”], prevents early disclosure of GBV”* (non-HCP, FG1). Again, confidence in the legal and healthcare systems are the key foundations for early disclosure and help seeking, regardless of the political status.

Participants repeatedly underlined the importance of confidence in the host country’s legal system, and that confidence needs to be established from the first contact with governmental institutions. Officers who carry out the first interviews with asylum seekers systematically screen for human trafficking. They have to distinguish true from falsely reported GBV as there is also a risk that *“some asylum seekers falsely report GBV experience to get political status”* (non-HCP, FG1). This creates an ambiguous situation for first interviewers between building confidence and uncovering falsely claimed violence. Other types of GBV have to be reported by the victims themselves. This often leads to a delay of disclosure.

Asylum seekers interact with the healthcare system only after the first interview. They receive medical assistance when there is a need. Building confidence in the healthcare system requires time and follow-up visits with the physician. One physician stated that *“it’s not during the first visit, but after three or four months that I follow them during pregnancy, that they start to have confidence and to talk about their experience”* (HCP, FG5). Continuity of care would therefore be of fundamental importance for a trust building process between the GBV victim and the caregiver.

During the focus groups and interviews, study participants discussed the need of a professional figure that is able to build trust with the asylum seekers and to establish their confidence with the legal and healthcare systems. This would eventually encourage GBV disclosure and help ensure continuity of care beyond cantonal borders. The description of this figure was most similar to the first-contact caregiver who was already working in federal accommodation centers. Indeed, the interview with the first-contact caregiver was the longest and provided much valuable information about all types of GBV, the asylum process, and networking among involved healthcare and non-healthcare professionals. This first-contact caregiver stated that a successful trust building process required time, presence, and active engagement with the asylum seekers.

### Communication, language and intercultural interpreters

Many participants agreed that confidence and GBV disclosure were strongly related to the ability to communicate one’s concerns. The existence of a language barrier by the asylum seeker who lack competence in the local language was very clear to the interviewees. Furthermore, participants also reported that victims may have a different way of expressing themselves. One participant relayed, *“she [a female asylum seeker from a sub-Saharan country] said she had a stomachache, but, rather, she meant that she was pregnant”* (HCP, I1)*.* This would lead to misunderstandings during medical visits and, possibly adverse events.

Related to this was the lack of language competencies to seek care for themselves and the need to use translators. Some participants stated that victims seek the aid of younger family members who may be more adept in the local language, which also means that the latter are faced with awkward situations. As an example, one participant cited a minor who reported that *“Mother says there is no blood”* which was supposed to mean that her mother was pregnant (HCP, FG5). Regarding language dissonance between care receiver and care provider, another participant revealed the lack of knowledge among professionals in the hospitals concerning how to seek language support:

“There is a lack of knowledge [among health professionals] about how to call a translator or an intercultural interpreter. So sometimes they use phone services. In emergency situations, patients use internet translators [Google translator]” (non-HCP, FG3).

The use of internet translators or hospital employees were often alternatives to intercultural interpreters.

In hospital settings, there is a list with employees who speak foreign languages. However, the presence of a man from the same culture in a labor room leads to uncomfortable situations. For example, a healthcare professional (FG5) said *“you cannot send a technician to the delivery room to translate. When she [the asylum seeker] is in labor and there is this man, she does not want to open her legs. I have to force her to give birth … It’s … like another violence to her”*. Supporting the need for female interpreters, as a competency that is available in the healthcare system, another healthcare professional participant from the same focus group further added: “*I sent a couple with domestic violence history to a physician who was from the same country and spoke the language. But the physician took the side of the man! He basically approved the violence!”*. Although a male interpreter or health worker from the same cultural background could represent a valuable resource, there is still, in many countries of origin, disparity between men and women. Having a male interpreter may thus not be beneficial for the GBV victim and could create further mistrust in the legal and health systems of the host country. Participants therefore underlined the importance and the need of female interpreters.

Financial support for the interpreters was not clear, which raises doubts about their availability as *“not all the professionals know who pays them [the interpreters]. Now they often use a 24-hour phone service. But we need interpreters*” (non-HCP, I2). Further, it was reported that intercultural interpreters can represent a bridge to address the language barrier and that they can be an aide in the trust building process between doctor and patient. Importantly, it was also underlined that the interpreter must be able to disappear from this triad and help to establish a trustful relationship. The interpreter should be a voice that translates but also interprets based on his or her knowledge of the culture. A participant highlighted that *“[an interpreter is] like a voice, [he/she does] translate what they tell [him/her] but can also interpret because [he/she] knows the culture behind [the words]”* (non-HCP, I2). This point had been further elaborated by the same participant adding that *“it’s not enough to translate, you need somebody who is able to interpret”*. This underlines the importance of a deeper understanding through a (female) interpreter instead of translation by electronic devices or translators.

### Continuity of care

As most asylum seekers were transferred from the federal asylum center to another cantonal accommodation within three months, participants expressed the challenge of building trust in such a provisional situation. In their case, a large diversity between the cantonal healthcare systems impeded a uniform availability of healthcare. This diversity implied additional work to find appropriate care facilities to address GBV victims, highlighted in the description below.

“Sometimes I sit down with them and we do a research on the internet together to find the right professional support in the canton they are transferred to. But there is a lack of continuity and of information inside the system” (non-HCP, I3).

Continuity of care was also disrupted by lack of transmission of paper-based information, which meant that the victim must disclose herself all over again for a second time. The participant continued *“there should be (although there is much sensibility about data security) transmission of information from one accommodation center to another. The NGOs in the new canton should receive information of a GBV victim to offer good treatment and protection. There should be a network”* (non-HCP, I3). Such lack of continuity, in the interviewees’ opinions, leads to lack of confidence between the victim and the system, and it also renews exposure to the trauma of telling the details of GBV. An example given by one of the participants highlights how this continuity of care was harmed. One participant specifically cited as an example a pregnant asylum seeker who was transferred to another canton during her third trimester:

“I followed her during the pregnancy, and we established a good patient-doctor relationship. Then she was transferred to another canton just two days before delivery. She had to adapt to a completely new environment with different people and a different language – why couldn’t they transfer her after the delivery?” (HCP, FG5).

Time is needed to build confidence in the system and in the healthcare professional. Once that is built, actions that result in change of healthcare professionals are counterintuitive. Pregnant asylum seekers represent an even more vulnerable group among GBV victims and should be granted a special protection during pregnancy and maternity. This would strengthen confidence in the legal and health systems.

### Sociocultural background and coping strategy

Participants felt that although the healthcare system offers several services to the victims, the moment of disclosure depends on individual factors such as sociocultural background, knowledge about GBV, stigma, coping strategy, and legal protection. The diversity of sociocultural backgrounds of women asylum seekers shows different coping strategy patterns. For example, they stated that some migrants, mostly Syrian women, are embedded in a strong family or larger social structure that offers a parallel care system. Their coping strategy is to talk about their experiences with other female family members. In contrast, young Eritrean migrants seek coping strategies within peers, as they may not have a family or social support. As one participant stated: *“Syrian women have their “clan” - they are embedded in a structure. They help each other. Eritreans instead, as we notice, are scattered” (HCP, FG2).* The lack of a larger network for Eritreans, as one participant pointed out, is because they escape their country due to its political system that forces young Eritreans to enroll in mandatory military service. However, *“Eritrean girls know what happens on the route. But they have no (life-) experience. They think somebody [Allah] will protect them, it would not happen to them. They are naive when they leave their country”* (HCP, I1). Thus, Eritrean asylum seekers are very young and have few life-experiences; they leave their home country alone or in small groups, which puts them at major risk of GBV on the route. This further leads to fewer social networks in the host nation.

Study participants reported that fears of stigmatization by peers from the home country and the victim’s sociocultural background influenced their willingness to disclose their GBV experience and delay seeking help. Many asylum seekers refuse psychological care because their peers could stigmatize them for it. They thus prefer to avoid talking about their traumatic experiences. A participant revealed that *“in their country, if you see a psychologist, you have a problem in your head. That’s why they don’t want to seek psychological support. That’s very stigmatizing…”* (non-HCP, I2). Another participant reported GBV victims’ avoidance: *“They say: I’m in Switzerland now, I don’t want to speak about these things, I just want to forget”* (HCP, FG5)*.* Participants agreed that active work on cultural approach and confidence at community level needs to be addressed to overcome the sociocultural barrier.

## Discussion

From our study findings, it is clear that lack of confidence in the legal and the healthcare systems is a major barrier and therefore, building confidence must be of the highest priority. The legal system must ensure credibility and strengthen victims’ rights. The healthcare system must offer a high standard of professionalism and ethics. Only then will the GBV victim be able to trust and overcome fear of stigmatization in order to disclose necessary information. Participants further noted linguistic competence as a barrier to communicate GBV experiences. They underlined the important role others play (younger family members and interpreters) in overcoming this barrier. Finally, an issue that study participants highlighted as very relevant to the legal and the healthcare systems was the transfer of asylum seekers from federal asylum centers to cantonal accommodations which disrupts the continuity of care. This disruption makes GBV an issue that risks not being raised or evaluated or being simply forgotten altogether.

In Switzerland the legal and organizational context applicable to asylum seekers who are victims of GBV offers only partial protection [13, 28]. This is evident as our study participants underscored that GBV victims disclose what happened to them only if the GBV experience could positively change their right to asylum. As a consequence, women who are victims of GBV on route would not disclose this issue because the stigma and fears related to this traumatic experience are higher and stronger than any willingness to talk about it. Information and trust enhancing strategies, including laws providing protection in these situations, would strengthen confidence among GBV victims. In line with our findings, it is also highlighted in the literature that victims do not disclose GBV on route because their aim is to reach their destination as soon as possible [42, 43]. For this reason, there is a need for mechanisms in the host countries to detect and address GBV in a timely, sensitive, and confidential manner. In Switzerland, professionals who carry out the initial asylum interviews do screen for specific GBV such as human trafficking. However, victims of other kinds of GBV must disclose themselves. The moment of disclosure is subjective and depends on the confidence the victims have in the legal and healthcare systems. For this reason, a systematic screening in different phases of the asylum process could give the opportunity to the GBV victims to disclose their experiences at the opportune moment. Our study participants emphasized that victims are primarily concerned about their asylum status and thus do not see the significance of disclosing their GBV experience during their asylum process. As victims are more likely to seek services from relevant healthcare institutions once the asylum status is confirmed, a rapid asylum process is therefore helpful.

Access to healthcare in the Swiss asylum context does not provide specific psychological support because of the brevity of the stay in federal asylum centers. This kind of support is offered once the asylum seeker has been transferred to a cantonal accommodation where long-term treatment can be initiated [32]. However, GBV victims need an early multi-faceted approach to address physical and psychological consequences of GBV [40, 42, 43]. Our study points to the important role that the first-contact caregiver can play in federal asylum centers. These professionals are in the best position to build trust on an individual level. For this reason, it could represent a first moment for disclosure and identification of GBV victims. In this case, the first-contact caregiver can act as a “GBV coordinator” [42, 43]. It is also important to guarantee financial support for such professionals and appropriate structures for confidential GBV screening approaches.

Similar to other studies, our participants also identified language as a main barrier to access healthcare [20, 21, 24, 38]. Misunderstandings in communication (e.g. “stomachache” meaning pregnancy), as well as use of family members or co-nationals as translators (e.g. family member, hospital staff) reveal the value of providing well-trained intercultural interpreters. Although interpreters reduce communication barriers, they represent a third person hindering the doctor-patient trust building process. Concomitantly, female interpreters might be more suitable to handle cases of female victims [21, 41]. In 2018, 60.5% of asylum seekers received asylum status in Switzerland [33–36]. Since it is likely that the majority of asylum seekers will remain in the country, language courses would enhance communication, and would promote integration and confidence in the Swiss authorities as well as in the healthcare system [33, 34]. As a consequence, GBV victims would be more prepared to disclose their experience and seek the care that they need. In this, our study’s findings are in line with other international reports [11]. Due to language barriers, trained and preferably female interpreters are indispensable. That these interpreters are much needed and critical in ensuring access to healthcare has been underlined in several Swiss [1, 18, 20, 24] as well as in international studies [21, 41, 49]. There is a clear need for good training of intercultural interpreters. Professional recognition would strengthen their position in the healthcare community. Ensuring a financial and legal framework that guarantees their independence is a prerequisite for allowing them to act as the true voice of the victim. Although there are different ongoing programs to reinforce the importance of intercultural interpreters [18], greater dissemination of information about intercultural interpreters among other health workers should be encouraged. Putting in place such measures and informing people widely about them (e.g. in leaflets in the native languages of the migrants) would add to the trust building process that is necessary in the healthcare setting. This is in line with the Swiss concept of access to healthcare in the asylum context [18, 33, 34].

On a structural level, lack of continuity of care represents a major issue in following and providing necessary healthcare when an asylum seeker is transferred from a federal asylum center to an accommodation in another canton. Although the process at the moment of transfer is clear, paper-based information does not seem to arrive at the destination. Instead, this lack of continuity provokes a loss of confidence in the system and the development of new self-protection coping strategies by the victim. The implementation of an electronic information exchange platform that is currently being spearheaded in the country could prove helpful for asylum seekers and the involved caregivers as it would enhance the seamless transmission of information and, thus, continuity of care.

Related to the transfer of asylum seekers to other cantons, those who are pregnant should benefit from special “maternity protection”. They might be offered the possibility to give birth in an environment where they have established a patient-doctor-relationship during pregnancy. This would strengthen bonding with the new-born, enhance confidence in the new state, and thereby favor better integration. In addition, financial support guaranteed by the involved authorities would help to build a network inside the healthcare system beyond the cantonal borders.

Furthermore, health professionals (general practitioners, gynecologists, pediatricians, psychiatrics, psychologists, nurses, and others) providing care to this group need to be aware of the sociocultural background of migrant populations, the different types of GBV, as well as the exposure risk in the home country, on the migration route, and in the host country. Hospitals, as the first care seeking point for migrants, offer special training to their professionals and have built a network of “Migrant friendly hospitals” [30], later named “Swiss Hospitals for Equity Network” [18]. In Switzerland, different universities and NGO’s offer courses in transcultural competence in health ([22]; Croix-Rouge [10]), which are good sources for health professionals to educate themselves to better care for their patients. On a primary care level, specific conferences about transcultural competencies and migrant health would be suitable to diffuse this knowledge to a broader population of healthcare professionals [24, 33, 34, 46]. A special certificate in transcultural medicine would underline the importance of the issue, give an incentive to health workers to attend the training, and enhance trust in the vulnerable population. Each canton could offer this training to professionals working in specialized centers caring for migrants. Additionally, the creation of a network of “Migrant friendly Doctors” or “Swiss Doctors for Equity Network” would ensure, among physicians in possession of such a certificate, a continuity of care that overcomes cantonal borders. Special reimbursement codes to reflect the more complicated and time-consuming care these populations need would underline these competencies and give additional incentives. Professionals with this special training could highlight their competencies and services to attract patients from this group [17, 20].

As described in different reports, a culturally appropriate multi-pronged approach should be applied in GBV prevention and response [42, 43]. In line with these reports, the findings of our research show that promotion of prevention strategies on an individual, community, and societal levels help strengthen resilience in the vulnerable population. These strategies also provide the opportunity to work with and educate potential perpetrators. This would help potential GBV victims to become more resilient, to know about their rights and to be informed about services that they can avail. Potential perpetrators must be made aware of the legal consequences of any form of GBV including sexual harassment, domestic violence, and traditional harmful practices. Integration programs would underline the importance of physical integrity and prevent gender-based violence.

In sum, these findings allow us to understand the actual context of the legal and healthcare systems, the barriers to healthcare access, as well as the strategic challenges. The federal and cantonal authorities should prepare themselves to ensure that those who are vulnerable and in need of care receive the necessary support from their host nation. Based on the findings and the discussion presented, we have summarized strategies that will improve GBV victims’ healthcare seeking behavior (Table 2). These strategies can also lead to better health outcomes, a decrease in preventable costs, including the higher costs related to the secondary morbidities that affect not only the women who have faced GBV but also their dependent children. Moreover, efficient measures would empower GBV victims, help them to become more resilient, and to potentially become promoters in the prevention of further GBV in and outside of Switzerland.

**Table 2:** Strategies to support victims of GBV

**1**	**Disclosure, identification and trust**	Implement different moments for screening procedures during the asylum process.
		Build confidence in the legal and healthcare systems through the role of a first-contact caregiver. This professional should build trust to encourage disclosure, ensure access to healthcare, and coordinate continuity at the moment of transfer. Systematically introducing this professional in all federal asylum centers would be advisable.
**2**	**Legal protection and prevention measures**	Engage leadership, strengthen legal protection, and offer judicial protection services.
		Educate GBV victims, potential perpetrators and the wider asylum-seeking population about local laws.
		Put in place prevention measures at the individual, community, and societal levels. Organize workshops, training, and information sessions on how to bolster resilience. Engage all the stakeholders involved in the asylum process.
**3**	**Training and financial support for healthcare workers**	Enhance knowledge about sociocultural background of different migration groups and provide adequate training and certification of health workers (e.g. “Swiss Doctors for Equity Network”).
		Guarantee financial support to intercultural interpreters.
**4**	**Financial support**	Dedicate financial support (cantonal and federal) that would help all the implicated professional groups (medical, paramedical, interpreters, NGOs, GBV Coordinator).
**5**	**Continuity of care**	Build a network of competent professionals and build an electronic information exchange platform to ensure a seamless continuity of care beyond cantonal borders.

### Limitations

It is important to note that the results of this qualitative study are not generalizable to the experiences of stakeholders working within the asylum system or the diversity that exists among asylum seekers in the country. Furthermore, the participants included in the research are professionals and not GBV victims themselves. Although there is much value to obtaining information from persons directly involved and with close experience, in light of the study goal and the barriers associated with studying GBV victims (e.g. lack of direct benefit, language, recruitment challenges), we found professionals to be an appropriate choice. GBV victims, who are in the asylum process, may not disclose their experiences easily and they might not agree to talk about this particular subject. Importantly, it is ethically difficult to justify a revival of their trauma for research purposes. In the future, further studies to capture GBV victims’ perspectives could be considered after cautious ethical approval. We did not delve deeper on legal aspects, because this study aims to investigate the actual situation and eventual gaps in the healthcare system, not the legal system. Finally, the small sample size could be an additional limitation, but this is due to the exploratory nature of the study and to the recruitment of the most important representatives from every step of the asylum process.

## Conclusions

In accordance with its international obligations derived from the 1951 Convention on the Status of Refugees and from International Human Rights law, Switzerland has the obligation to guarantee basic financial (social aid or urgent aid) and healthcare services (art. 80 Asylum Act). In addition, healthcare workers have professional and ethical obligations to provide adequate care for vulnerable groups independently of their legal status (resident, refugee, asylum seeker, prisoner, etc.). The healthcare system is tasked with ensuring a good quality of care to these residents. A knowledgeable and well-prepared healthcare system is important to provide much needed care for GBV victims, starting from their first disclosure to specific treatments that begin on their arrival and continue until their final destination. It is important to establish an appropriate organizational framework that protects the victims and works to ensure their best health outcomes. This includes a healthcare system with competent professionals and a seamless continuity of care beyond cantonal borders. Strengthening integration services and programs available for asylum seekers would help them enforce their capability to address their own care needs. Finally, it is important to put in place early interventions to decrease long-term consequences of GBV because doing so will have positive outcomes not only for the individual concerned and her family, but also for the reduction of costs to the entire healthcare system. Further research is needed to establish ethical and professional guidelines.

## Data Availability

The data generated and/or analyzed during the current study are available from the corresponding author upon reasonable request provided it does not breach the confidentiality agreement.

## Abbreviations

GBV: Gender-based violence
CEDAW: Convention on the Elimination of Discrimination against Women
WHO: World Health Organization
UNHCR: United Nations High Commissioner for Refugees
UNFPA: United Nations Population Fund, formerly United Nations Fund for Population Activities
WRC: Women’s Refugee Commission
IOM: International Organization for Migration
PTSS: Post-traumatic stress syndrome
OHCHR: Office of the High Commissioner of Human Rights
NGO: Non-Governmental Organization
FOPH: Swiss Federal Office of Public Health
FG: Focus-groups
I: Interview
HCP: Healthcare professional
non-HCP: Non-Healthcare professional
UMC: Ufficio del Medico Cantonale (Office of the Chief Medical Officer for the Canton)
HUG: Hôpitaux Universitaires de Genève (Geneva’s University Hospitals)
EHR: Electronic Health Record
